# Supplementary material for: Patterns of Intron Gain and Loss in Fungi
Source: PLoS Biol. 2004 Nov 30;2(12):e422. doi: 10.1371/journal.pbio.0020422 (PMC532390; doi:10.1371/journal.pbio.0020422)
Supplement: Table S1 — Also available at http://genes.mit.edu/NielsenEtAl/. (4.3 MB ZIP). [file pbio.0020422.st001.zip › NielsenEtAl/html/440.html]

AN2439.1.NCU09744.1.MG06111.1.FG06219.1


```
 CLUSTAL W (1.82) Multiple Sequence Alignments - Introns Inserted


Sequence 1: NCU09744.1	353 aa
Sequence 2: FG06219.1	348 aa
Sequence 3: AN2439.1	339 aa
Sequence 4: MG06111.1	352 aa
Alignment Length: 365 aa
Number Identitical Residues: 120 aa
Alignment Score (without introns) 7184


MG06111.1 	----~-MFEAEPAPGDCPTAMKFAP-GSRKLLVSSMDGNIYMYELQGE-GEDASAPLVRQ
NCU09744.1	MAPS1TQFEVSQPPSDAISAVIFAPDNTNRLLVSSWDRNVYLYEIAEG---TEDATLINK
FG06219.1 	MAPA1TQYELSPSPTDAVSSIAFAPSSGTKLLVSSWDKKVYCYDIAGG---AGETTLINT
AN2439.1  	----~--MKFSPAP------------DSTRFVVSSWDKNVYVYDLRDENGAAGEGKLLQK
          	        : . .*            .  :::*** * ::* *::   ..   .  *:. 

MG06111.1 	ISIGCPVLDVTFGSDDKEGFCTGADSAIKR2VDLESGDVTVVGKHEKPARCIIYSPEYS1
NCU09744.1	FEHRAPVLDVCFGANGNEAYTASLDCSVKR2IDLSTGEQTVLSTHEKPVRCVVYSPQHS1
FG06219.1 	YEHRAPVLDVCFGANDNEAFTAGMDWAVNK2IDLETGEKTQLSKHAAPVRSIAYSPTFS1
AN2439.1  	FEHRAPVLDACFGATEDEIFTAGLDWDVKR2IDIASASQTVLSSHDAGVRSVVYSKEYS~
          	 .  .****. **:  .* : :. *  ::: :*: :.. * :..*   .*.: **  .* 

MG06111.1 	ILASGSWDCTLQIWNAKDLSKDP--IIVQLPVKVHAMAASKTKLVVGMHNRMVQIFDLPA
NCU09744.1	MLISASWDHTLHVHNTSAPASTP--ITISLPGKPHALAASPSKVVVAMTARLVHIYDLPT
FG06219.1 	ILVSASWDCSLNLHNLNDPSSTP--IRVSLPGKPHALAASPTKIVVAMAGRVINIYDLKT
AN2439.1  	MVISASWDNTLHVHRLAGDRSVTNTASIPLPSKPFSMSLTATKLVVGMASRALHIYDLKS
          	:: *.*** :*:: .     . ..:  : ** * .::: : :*:**.*  * ::*:** :

MG06111.1 	IAQLLESGAS---GSESGLKPWQQRESSLKFMTRAIACMPNDAGYATSSTEGRVAVEFFE
NCU09744.1	LATALSSAD----SASANITPWQQRESSLKFLTRAVACMPNDAGYATSSIEGRVAVEWFE
FG06219.1 	IADLFATG-------SSDLQPWQQRESSLRYLTRAVSCMPNDAGYATSSIEGRVAVEWFE
AN2439.1  	LSLLTGQSEGGVAPAKVEVEPWQRRESSLKFMTRCVACMPDDAGYASSSIEGRVAVEWFD
          	::     . .. : :.  : ***:*****:::**.::***:*****:** *******:*:

MG06111.1 	DSAEVQARKYAFKCHRGPDPKD-PDTELIYPVDSLAFHPEYLTFVSGGGDGQVALWDSEA
NCU09744.1	DTAESQARKYAFKCHRQTAPEEEGGGDVVYPVNALTFHPVYGTFASGGGDGTVALWDAEA
FG06219.1 	DTPESQARKYAFKCHRQAAPD--GDGDIVYPVNALAFHPVHGTFASGGGDGTAALWDAEA
AN2439.1  	PSAESQARKYAFKCHRQTSDD----VDVVYPVNSLAFHPIHGTFASGGGDGVVALWDGIA
          	 :.* *********** .  .     :::***::*:*** : **.****** .****. *

MG06111.1 	KRRMKIYPMNGGLAARTLAFSADGRFLAIGTCPGFEDTMENYSGKGQSHVLIRELSEKEV
NCU09744.1	KRRMRQYQKFPDSVA-ALAFSNNGKYLAIGVCRGFETDMEDFNSEGRTKVFIRELGETEA
FG06219.1 	KRRLKQYQKFPNSVA-ALSFSSDGKYLAVGVCPGFETGQEDYSGAGQTSVLIRELGENEA
AN2439.1  	KRRIRQYQKYPSSVA-AVAFSSNGKYLAIAISPGYEDGKDDLT-DGTVRIYVRELGETEA
          	***:: *    . .* :::** :*::**:. . *:*   :: .  *   : :***.*.*.

MG06111.1 	KPKPKKSK
NCU09744.1	KGKGPK--
FG06219.1 	KGKGAK--
AN2439.1  	KGKGAK--
          	* *  *
```
